# Review of "What bugged the dinosaurs? Insects, Disease and Death in the Cretaceous" by Poinar G. Jr. and Poinar R

**DOI:** 10.1186/1756-3305-1-6

**Published:** 2008-03-16

**Authors:** Raymond L Jacobson

**Affiliations:** 1 Department of Parasitology, The Hebrew University-Hadassah Medical School, POB 12272, Jerusalem, 91120, Israel

## Review

Have you ever wondered whatever happened to the dinosaurs? George and Roberta Poinar have put forward some evidence that maybe it was not just cataclysmic events, such as meteorites falling on the earth. They surmise that perhaps insects transmitted diseases that contributed to the extinction of the dinosaurs. By studying the arthropods trapped in amber during the Cretaceous (65.5 – 145.5 million years ago) period, they have revealed some extraordinary micro-organisms concomitant with the ensnared invertebrates.

The period is well described in the opening chapters, showing that fossil evidence and especially amber tells us a great deal about the animal and plant kingdoms during those millions of years. Some chapters start with a speculative scene, painting a picture of life in the Cretaceous, the dinosaurs, the plants they feed from and the insects that breed around them, while others discuss in detail the known scientific facts. Herbivory, both by the dinosaurs and the insects is described in detail and the possibility that insects introduced plant viruses and fungi into the food supply, which may have led to the depletion in resources for the large animals. The dinosaurs did benefit from insects, like the dung beetles that removed the vast waste voided by 55–100 ton dinosaurs, and arthropods were part of the diet of the omnivores.

The authors describe how they believe that arthropods were able to acquire blood meals from the dinosaurs in antiquity. By studying the mouth parts of the insects trapped in amber, they have shown that regardless of the outer skin, whether cold or warm blooded, the micro-predators had found a way to obtain the necessary food for survival. Chapters 12 – 18 describe those blood-sucking arthropods that were extant during the Cretaceous, including, important Nematocera and Tabanids, fleas, lice, ticks and mites. For each group the method of haematophagy is discussed and which organisms could have been transmitted with a few examples of ancient parasites observed in amber. There are separate chapters on the worms, cretaceous diseases, and another on the evolution of pathogens, (erroneously Rickettsia are given as the cause of human plague). The numerous color plates illustrate the diversity of arthropods in the Cretaceous, while the original line drawings embellish the theory. This is an assiduously written book for entomologists and parasitologists who would like to lean more on the time-encapsulated data from the Cretaceous, and perhaps stimulate the search for more "paleoparasites".

## Competing interests

The author(s) declare that they have no competing interests.

